# Molecular Pathogenesis of Anti-NMDAR Encephalitis

**DOI:** 10.1155/2015/643409

**Published:** 2015-06-28

**Authors:** Hao Ding, Zhihong Jian, Creed M. Stary, Wei Yi, Xiaoxing Xiong

**Affiliations:** ^1^Department of Neurosurgery, Renmin Hospital of Wuhan University, Wuhan, Hubei 430060, China; ^2^Department of Anesthesiology, Perioperative and Pain Medicine, Stanford University School of Medicine, Stanford, CA 94305, USA; ^3^Central Laboratory, Renmin Hospital of Wuhan University, Wuhan, Hubei 430060, China

## Abstract

Anti-NMDAR encephalitis is a recently identified autoimmune disease, described by an immune-mediated loss of NMDA glutamate receptors, resulting in progressive mental deterioration. To date, literature on anti-NMDAR encephalitis has been largely clinically oriented, including descriptions of the clinical presentation and course, diagnostic methods, and potential clinical treatments. However, the underlying molecular mechanisms contributing to the complex immunological cellular transformation that is associated with the progression of anti-NMDAR encephalitis remain to be adequately explored. This review will provide a summary of the current literature on anti-NMDAR encephalitis, including the immunologic molecular mechanisms contributing to disease progression. In particular this review will focus on the effect of anti-NMDAR on GluN2-NMDAR expression and the molecular transformation of B and T leukocytes in the loss of self-tolerance. Further research on the immunologic mechanisms contributing to anti-NMDAR encephalitis may provide an avenue for future novel diagnostic approaches, such as immunologic surveillance, as well as new therapeutic strategies for this recently identified autoimmune disease.

## 1. Introduction: Anti-NMDAR Encephalitis

*Autoimmune Encephalitis.* In 2004, encephalitis of unknown etiology primarily affecting young women presenting with the onset of acute behavioral disturbances was first recognized in Japan [1]. Subsequently, in 2005, four young women with ovarian teratomas were observed with similar states of agitation and psychosis [2]. With no clear nomenclature for this encephalitis, the syndrome received many names, including acute reversible limbic encephalitis [3], acute juvenile female nonherpetic encephalitis [1], and juvenile acute nonherpetic encephalitis [4]. With the cooccurrence of teratoma, and clinical improvement following teratoma removal, the syndrome was considered paraneoplastic [5]. However, the subsequent detection of antibodies to synaptic proteins [6] provided evidence of a coexisting immune-mediated pathogenesis, more appropriately categorizing the syndrome as an autoimmune encephalitis.

The clinical presentation of autoimmune encephalitis varies; however, patients generally express a viral prodrome, followed by the development of acute psychiatric symptoms, memory problems, seizures, decreased or confused consciousness, and dyskinesias [7]. Neurologic symptoms (e.g., dyskinesias and seizures) tend to be the initial clinical manifestation for younger patients (≤18 years) [8]. Older patients (≥45 years) tend to present with memory loss, making differentiation from other dementia-associated disorders difficult [9]. However, a significant clinical tool for diagnosis in women continues to be the presence of tumors: approximately 45% of patients older than 18 years and 9% of girls younger than 14 years present with ovarian teratomas [10]. Autoimmune encephalitis can therefore be broadly divided into two categories: classic tumor-associated paraneoplastic disorders (PNDs) and tumor-absent disorders associated with antibodies to neuronal cell-surface or synaptic receptors [11]. The PNDs are relatively rare and in most cases affect older women [12]. Patients with PNDs generally experience a monophasic clinical course and exhibit better response to treatment if the disorder is recognized early and the tumor removed, while autoimmune encephalitis occurring in the absence of tumor has a more variable response to treatment [13].

*Identification of Anti-NMDAR Encephalitis Subtype.* Antibodies to multiple synaptic targets have been identified in patients with symptoms of encephalitis, including the glutamate receptors GluA1 and GluA2, subunits of the alpha-amino-3-hydroxy-5-Methyl-4-isoxazolepropionic acid receptor (AMPAR) [14], the leucine-rich glioma-inactivated 1 protein (LGI1) [15], the B1 subunit of the -aminobutyric acid-B receptor (GABABR) [16], and the metabotropic glutamate receptor 5 [17]. However, the most common form of autoimmune encephalitis with loss of self-tolerance to synaptic proteins occurs with detectable autoantibodies against the N-methyl-D-aspartate receptor (NMDAR) [18, 19]. Autoantibodies directed against the NR1 subunit of the NMDAR are thought to be responsible for the neurobehavioral pathology [5]. These autoantibodies have been shown to result in a decrease in the number of NMDARs in target cells by inducing crosslinking and internalization of NMDARs by autophagy [20]. This form of the disease has now been officially categorized, termed “anti-NMDAR encephalitis” by Dalmau and colleagues in 2007 [5].

*Epidemiology and Pathology.* The exact incidence of anti-NMDAR encephalitis is unknown. However, estimates place 20% of patients with autoimmune encephalitis with detectable levels of antibodies to NMDAR [21], exceeding the prevalence of enterovirus or herpes simplex virus-1 (HSV-1) encephalitis in young adults [22]. In one case series [14], 77 of 100 patients presenting with signs of encephalitis and psychiatric symptoms tested positive for anti-NMDAR antibodies. Increasing numbers of case reports and evidence from intensive care [23] and pediatric patients [24] suggest that anti-NMDAR encephalitis may be more frequent than any other known paraneoplastic encephalitis. A multicenter, population-based prospective study [25] suggested that anti-NMDAR encephalitis accounts for 4% of all causes of encephalitis. After acute disseminated encephalomyelitis, the disorder was the second most common immune-mediated encephalitis, confirmed in 20% of cases of encephalitis at one center using retrospective analysis of serum [21]. These findings suggest that anti-NMDAR encephalitis is not rare and likely commonly misdiagnosed as a seizure disorder or psychiatric illness [18]. However, psychiatrists now recognize this syndrome as a distinct disease that should be identified from other cases of first episode psychosis [26, 27].

NMDARs play a key role in several synaptic adaptation processes and NMDAR signaling dysfunction [28]. For example, NMDAR overactivity induces excitotoxic postsynaptic neuronal cell death and persistent upregulation of AMPAR function, leading to alterations in hippocampal long-term potentiation [28]. NMDAR overactivity is the proposed underlying mechanism in epilepsy, dementia, and stroke, whereas decreased NMDAR activity results in symptoms of schizophrenia [29].

## 2. NMDAR: Structure

### 2.1. Molecular Structure

Conventional NMDAR are tetrameric complexes composed of glycine-binding NR1 subunits and glutamate-binding NR2 (NR2A–NR2D) subunits [30]. These subunits assemble together to form receptor subtypes with distinct synaptic localization, physiological and pharmacological properties, and intracellular signaling properties. Localized in postsynaptic membranes, these receptors serve as ligand-gated cation channels which function in synaptic transmission and plasticity [31].

### 2.2. Anti-NMDAR Antibody and NMDAR Trafficking

GluN2A-NMDAR and GluN2B-NMDAR subtypes expressed in hippocampal neurons [32] are exclusively located in the postsynaptic membrane compartment [33]. While both GluN2A-NMDAR and GluN2B-NMDAR are detected at the neuronal surface, GluN2A-NMDAR has a clear enrichment in the postsynaptic density of glutamatergic synapses. GluN2B-NMDAR is mostly extrasynaptic [34]. IgG autoantibodies to NMDAR are the only serotype specific to anti-NMDA encephalitis, and levels correlate with neuropsychiatric symptoms in a titer-dependent manner [19]. Application of anti-NMDAR IgG isolated from patient CSF to neurons in culture rapidly reduced GluN2A-NMDAR (synaptic) and GluN2B-NMDAR (extrasynaptic) surface content and reduced potentiation of glutamatergic synapses [35]. However trafficking of other membrane receptors and channels remained mostly unaffected [35].

The surface distribution of NMDAR is determined by receptor diffusion in the plasma membrane [36]. The diffusion of mobile surface GluN2A-NMDAR and GluN2B-NMDAR was differentially affected by application of patient CSF IgG whereby patient CSF preferentially increased the mobile diffusion of GluN2A-NMDAR. The proposed molecular mechanism is as follows: patient IgG induces a rapid dispersal of GluN2A-NMDAR, preventing dynamic synaptic retention and inhibiting the downstream interaction between extracellular domains of GluN2A-NMDAR subunits [37]. While the mobile fraction of GluN2B-NMDAR was mostly removed from synapses, extrasynaptic GluN2B-NMDAR was found to be mostly cross-linked and internalized by autophagy [37].

### 2.3. The Interaction between GluN2A-NMDAR and EPHB2R Is Disrupted by Anti-NMDAR IgG

Numerous ligands interact with the intracellular domain of the GluN2A subunits of NMDAR [32]. However, few ligands for the extracellular domain of GluN2A subunits have been identified. Among those extracellular ligands identified, the ephrin B2 receptor (EPHB2R) efficiently clusters NMDAR when activated by ephrin B2 ligand [38]. Mikasova and colleagues [35] demonstrated that the interaction between EPHB2R and GluN2A subunits of NMDAR is disrupted by application of patient IgG, resulting in a lateral dispersal of synaptic EPHB2R and NMDAR.

Interestingly, this effect can be reversed by coapplication of ephrin B2, preventing the increased surface diffusion and lateral escape of synaptic GluN2A-NMDAR and effectively rescuing cells from IgG-mediated cell death. This effect appears to be specific to EPHB2R as activation of the indirect NMDAR activator N-cadherin receptor [39] did not affect patient IgG-induced lateral redistribution of synaptic GluN2A-NMDAR. Therefore, excitotoxicity and neurobehavioral sequelae secondary to anti-NMDAR IgG appear to be the result of disruption in the interaction between EPHB2R and NMDAR, leading to dysregulation of synaptic retention of GluN2A-NMDAR and neuronal cell death.

## 3. Autoimmunization: Mechanisms Leading to Loss of Self-Tolerance

### 3.1. B and T Leukocyte Transformation

The association between tumor occurrence and autoimmunity has been explored. One proposal is that the presence of tumor provides a source of an unknown self-antigen, leading to expansion of T and B leukocyte cells and tumor-specific antibodies, ultimately resulting in cross-reactivity with NMDARs [40]; because there is a strong correlation between excitotoxic cell death, mental dysfunction, and increased calcium influx [41, 42], it has been proposed that circulating antibodies and cytokines cross the blood brain barrier, modulate NR2A and NR2B subunits in the hippocampus and neocortex of brain, and increase calcium conductance. The primary activator of B and T leukocytes is the antigen presenting cell (APC) [43]. APCs presenting a self-tumor antigen in the presence of a costimulatory signal in the form of upregulated CD80 or CD86 (normally triggered by a microbial stimulus) induce either anergy, deletion of tumor-specific T cells, or expansion of T-regulatory cells [43]. Having a similar mechanism, B leukocytes also undergo apoptosis or anergy when they encounter a self-antigen presented by APC in the absence of T leukocyte help [44]. In addition, the tumor antigen itself is tolerogenic [45]: immunosuppressive cytokines (IL-10 and TGF-b) produced by tumors suppress expansion and differentiation and induce apoptosis of T leukocytes. The underlying mechanism for this may also be related to tumorigenic activation of indolamine-2-3-dioxygenase, which rapidly consumes tryptophan, thereby preventing T leukocyte expansion [45].

### 3.2. Costimulation by Pathogens May Induce Anti-NMDAR Autoimmunity

Nearly fifty percent of anti-NMDAR encephalitis patients have no detectable tumor. In these patients, pathogenic infection may provide an antigenic trigger for development of loss of self-tolerance [46]. This process has been observed in rheumatic fever, whereby arthropathies and cardiac valvulopathies develop secondary to cross-reactivity with peripheral connective tissue [47]. Primary biliary cirrhosis may also be associated with microbial infection [48].

Numerous pathogens, including Mycoplasma pneumonia [6] and Epstein-Barr virus [49], have been detected in the serum of affected patients. The postulation has therefore been put forward that pathogens provide a costimulus inducing autoimmunity. Furthermore, endogenous retroviruses, which account for 1–8% of the human genome, may contribute to autoimmunity [50] as they are commonly detected in teratomas [51]. This is supported by observations of endogenous retroviruses reactivation in some autoimmune diseases and cancers [51]. Coactivation by pathogen adjuvants in the development of autoimmunity has been described in other disorders [52].

### 3.3. The Innate Immune System Regulates Adaptive Autoimmunity

Tumors are potential sources of ligands for innate immune system pattern recognition receptor proteins, such as toll-like receptors (TLRs), nucleotide oligomerization domain- (NOD-) like receptors, retinoic acid-inducible gene-1 (RIG-1), and melanoma differentiation-associated protein-5 (MDA-5) helicases [53]. Upregulation of the major histocompatibility complex (MHC) Class I and Class II receptors, along with alterations in CD80 and CD86 expression on APCs, can result in breakdown of T cell tolerance mechanisms and the generation of autoimmunity [53]. Autoreactive T cells induce somatic hypermutation of B cells (via CD40-CD40L interactions) and generation of a tumor-specific response. Tumor-specific B cells induce class-switching recombination (CSR) leading to antibody diversification and affinity maturation during autoimmunity. In CSR, with T-cell support, mature B lymphocytes switch from producing the *μ* chain of IgM to an alternate heavy chain: *γ* for IgG1, *α* for IgA, and *ε* for IgE [54]. In anti-NMDAR encephalitis, T lymphocytes and B lymphocytes immunotolerance is disrupted, as the specific antibodies detected in patients demonstrate class-switching to IgG1 and IgG3 [54].

### 3.4. Blood Brain Barrier Disruption Permits B Cell Infiltration

Activation of innate immune-mediated cytokines and TLR ligands leads to disruption of the blood brain barrier (BBB) [55], induced by activated cytokines and TLR ligands [55]. Additionally, IL-17 produced by autoreactive Th17 cells [56] can also influence the tight junctions between endothelial cells of the BBB. All of these factors contribute to BBB permeability, allowing infiltration of autoreactive memory B lymphocytes and other relative immunological cells. Under the effect of the B cell activation factor (BAFF), autoreactive B cells undergo expansion [56], leading to production of specific immunoglobulin in patients. Rapid and substantial production of B-cell derived immunoglobulins may contribute to the rapid clinical deterioration observed following the prodromal phase, and the poorer response to therapeutic interventions in patients with high IgG titers.

### 3.5. Extra-CNS NMDA Receptor-Induced Autoimmunity

NMDARs are also located in several locations outside of the CNS, including kidney [57], lung [58], myocardium [59], lymphocytes [60], pancreatic beta cells [61], parathyroid glands [62], and megakaryocytes [63]. Owing to the mechanisms of self-tolerance discussed above, these receptors do not normally exhibit auto-autoimmunity. However, case reports in swine abattoir workers with monophasic inflammatory polyradiculopathy following exposure to aerosolized brain tissue demonstrated levels of serum IgG reactive to mouse brain tissue [64]. This suggests that cross-reactivity to exogenous NMDAR may contribute to loss of autoimmunity when presented at respiratory or intestinal surfaces or in lesioned tissue.

## 4. Immunotherapy and Treatment Outcomes

To date, effective pharmaceutical strategies aimed at preventing NMDAR dysfunction are unsatisfactory, as most drugs are not specific for NMDAR subtypes [65]. However several promising therapies have provided a beneficial outcome. Once the diagnosis of anti-NMDAR encephalitis is confirmed, many patients are initially treated with first-line immunotherapy, including corticosteroids, intravenous immunoglobulin, plasma exchange, and/or anti-inflammatory agents [18]. However, these approaches are not effective at decreasing intrathecal antibody titers. Patients who did not respond to first-line therapies may then undergo second-line immunotherapy. For example, monoclonal antibodies directed against CD-20 B lymphocytes (rituximab) may be used in sequence or in combination with cyclophosphamide [19].

Although no standard guide for immunotherapy exists, Dalmau and collogues [18] have found success with IV-Ig (0.4 g/kg per day for 5 days) and methylprednisolone (1 g/day for 5 days) to plasma exchange. They suggest initiating second-line therapy if no response is observed after 10 days. In adults, this consists of rituximab (375 mg/m^2^ every week for 4 weeks) combined with cyclophosphamide (750 mg/m^2^ given with the first dose of rituximab), followed by monthly cycles of cyclophosphamide. Antiepileptics are not needed in most patients. Because relapses occur in 20–25% of patients, often in those without teratoma, continued immune-suppression (mycophenolate mofetil or azathioprine) for at least 1 year after initial immunotherapies are discontinued is recommended. With this regimen, up to 75% of patients exhibit total or near-total recovery, while 25% of patients exhibit persistent severe neurological deficits or die [18]. Unfortunately the occurrence of disease relapse following the above treatment paradigm has not been reported [18]. However, the risk of relapse is significant if tumor is present and not recognized early in the course and treated appropriately [18].

## 5. Summary and Future Directions

Currently, identification of the disease remains limited by the lack of a rapid blood test for early diagnosis and initiation of therapy. Treating physicians with clinical suspicion for anti-NMDAR encephalitis in patients presenting with psychiatric symptoms are limited to diagnostic imaging [27], video electroencephalograms [66], and laboratory testing of CSF for IgG [19] to confirm diagnosis. While several lines of research have provided significant insight into the molecular pathogenesis of anti-NMDAR encephalitis including the transformation of B and T leukocyte, the mechanisms leading to loss of self-tolerance remain unclear. In addition to the development of an effective therapy aimed at preventing cross-reactivity in at-risk patients (e.g., those found to have teratoma), future investigations aimed at developing novel immunotherapies specific to anti-NMDAR IgG may provide benefit for those 25% of patients with poor response to current therapy.
